# Per-Event Probability of Hepatitis C Infection during Sharing of Injecting Equipment

**DOI:** 10.1371/journal.pone.0100749

**Published:** 2014-07-07

**Authors:** Lies Boelen, Suzy Teutsch, David P. Wilson, Kate Dolan, Greg J. Dore, Andrew R. Lloyd, Fabio Luciani, on behalf of the HITS investigators

**Affiliations:** 1 Inflammation and Infection Research Centre, School of Medical Sciences, The University of New South Wales, Sydney, Australia; 2 The Kirby Institute, The University of New South Wales, Sydney, Australia; 3 Section of Immunology, School of Medicine, Imperial College, London, United Kingdom; 4 National Drug and Alcohol Research Centre, The University of New South Wales, Sydney, Australia; University of Sydney, Australia

## Abstract

**Background:**

Shared injecting apparatus during drug use is the premier risk factor for hepatitis C virus (HCV) transmission.

**Aims:**

To estimate the per-event probability of HCV infection during a sharing event, and the transmission probability of HCV from contaminated injecting apparatus.

**Methods:**

Estimates were obtained using a maximum likelihood method with estimated IDU and sharing events obtained from behavioural data.

**Settings:**

Cohort study in multiple correction centres in New South Wales, Australia

**Participants:**

Subjects (N = 500) with a lifetime history of injecting drug use (IDU) who were followed up between 2005 and 2012. During follow-up, interviews for risk behaviours were taken and blood sampling (HCV-antibody and RNA testing) was performed.

**Measurements:**

Self-reported frequencies of injecting drugs and sharing events, as well as other risk behaviours and details on the nature of injecting events.

**Findings:**

The best estimate of the per-event probability of infection was 0.57% (CI: 0.32–1.05%). A sensitivity analysis on the likely effect of under-reporting of sharing of the injecting apparatus indicated that the per event infection probability may be as low as 0.17% (95% CI: 0.11%–0.25%). The transmission probability was similarly shown to range up to 6%, dependent on the presumed prevalence of the virus in injecting equipment.

**Conclusions:**

The transmission probability of HCV during a sharing event is small. Hence, strategies to reduce the frequency and sharing of injecting equipment are required, as well as interventions focused on decreasing the per event risk.

## Introduction

Approximately 130–170 million people worldwide have been infected with hepatitis C virus (HCV), with 3–4 million new infections annually [1]. For the 75% of those infected who develop chronic infection, slowly progressive fibrosis culminating in cirrhosis and ultimately liver failure and hepatocellular carcinoma may follow. Transmission is predominantly parenteral – in the developed world this is primarily associated with sharing and re-use of injecting apparatus amongst people who injecting drugs (PWID) [2]. The cumulative lifetime prevalence of HCV infection in PWID is up to 90%, dependent on the duration of injecting drug use (IDU) [3].

PWIDs have high rates of imprisonment due to the illegal nature of drug use, and the high rate of crime committed to fund drug dependence. For instance, almost half of Australian prisoners report a lifetime history of IDU and more than half are incarcerated for drug-related crimes [4], with similar rates in the USA [5]. Accordingly, HCV infection is very common in custodial populations, with a seroprevalence of up to 80% amongst imprisoned PWID in Australia, USA, and UK [4], [6]–[8].

To date, the per-event probability of HCV infection following an episode of sharing of an injecting apparatus remains unknown. This probability is dependent on multiple factors including: the availability of a clean and sterile injecting apparatus, and hence the likelihood of sharing and contamination of the apparatus with blood of the source(s); the prevalence of viraemia and the viral load in the source(s); and the capacity of the virus to survive *ex vivo* in the context of the injecting equipment. Transmission will also depend on preventative measures, such as the potentially virocidal effects of bleach cleansing of the injecting apparatus [9], [10].

In this analysis, a quantitative estimate of the per-event probability of HCV infection associated with an IDU event involving sharing of injecting equipment (needle, syringe and other paraphernalia listed in Table 1) within the custodial environment was calculated from the Hepatitis C Incidence and Transmission Study in prisons (HITS-p), which is a longitudinal cohort of uninfected prisoners who report IDU, with a previously reported HCV incidence of 34.2 per 100 person years [11]. The estimates of the per-event probability of infection mirror the inherent uncertainty regarding the presence of HCV-contamination of the injecting equipment used during a sharing event. In addition, we also estimated the transmission probability, which assumes that the injecting apparatus is indeed infected with HCV.

**Table 1:** Demographic characteristics and injecting behaviours in the two study cohorts.

Subjects characteristics	Prospective	Retrospective
	N = 92	N = 106
Age	25 (18–39)	24 (18–48)
Male	64 (70%)	75 (71%)
Duration of IDU in years: median (range)	6 (1–22)	6 (0–34)
Injecting heroin	64 (70%)	59 (56%)
Injecting methamphetamines	60 (65%)	43(41%)
Receiving MMT at enrolment	21 (23%)	26 (25%)
Mean number of IDU events per year	199 (1–572)	83 (6–572)
Reported sharing	58 (63%)	77 (73%)
Reported sharing of syringe/needle	55 (95%)	76 (99%)
Reported sharing paraphernalia (any)	45 (78%)	58 (75%)
Reported sharing Spoon	41 (71%)	56 (73%)
Reported sharing Mix	27 (47%)	41 (53%)
Reported sharing Filter	29 (50%)	41 (53%)
Reported sharing Swab	3 (5%)	17 (22%)
Reported sharing Tourniquet	10 (17%)	20 (26%)
Reported sharing only after bleach cleansing	33 (57%)	63 (23%)
Mean number of sharing events per year^1^	56 (1–364)	47 (1–429)

1 Estimate based on intermediate estimate from categorical data available on IDU frequency, for subjects who reported sharing.

## Methods

### Subjects

The ongoing HITS-p cohort was established in 2005 within correctional centres in New South Wales (NSW), Australia [11]. Data were available for this analysis from 500 enrolled prisoners who reported a lifetime history of IDU and had a negative HCV antibody test within the preceding 12 months. These prisoners (N = 500) were followed up in 26 centres, with a minimum of 10 interviews performed in each site. At enrolment, after written consent, demographic data as well as detailed information regarding risk behaviours for HCV transmission were recorded by structured interview in relation to the period ‘since imprisonment’ (see details of the interview in [11]). In addition, HCV antibody and RNA testing were performed. Subjects were followed-up six-monthly whilst in prison recording risk behaviours ‘since your last interview’, as well as repeat antibody and RNA testing. Relevant institutional review board approvals were obtained. The study was approved by the Human Research Ethics Committees of Corrective Services New South Wales (No. 05/0884) and of Justice Health (No. GEN 31/05).

### Subject selection

Two analyses on the HITS-p study cohort were conducted. Firstly, data were analysed from subjects who had confirmed HCV-uninfected status at enrolment into HITS-p and who were followed prospectively for a period of at least two years (N = 92). Inclusion criteria for this analysis were subjects who: had a negative pre-enrolment HCV screening antibody test; negative HCV antibody and PCR tests at enrolment; and who reported at least one IDU event during prospective follow-up after enrolment. A second analysis was conducted on a retrospective dataset covering the period since imprisonment until enrolment in the cohort (N = 106). We included subjects who reported IDU between imprisonment and enrolment, with an HCV antibody negative test at enrolment and were in prison for less than a year, or had been in prison more than a year at enrolment but were HCV antibody negative during the year before enrolment. For those subjects (N = 40) who were in prison for longer than one year, we considered only risk behaviours during 1 year prior to enrolment. Although the prospective and retrospective datasets were mutually exclusive, there were 34 subjects who contributed data (from different time periods) to both analyses.

### Estimated time of infection

The estimated date of infection for each incident case was calculated using the following algorithm: for subjects who seroconverted during follow-up, the midpoint between the date of the last HCV antibody negative sample and the first positive was utilised; for subjects who were viraemic, but HCV antibody negative, at the initial detection timepoint the date of infection was taken as the date of the last test minus 51 days [12].

### Estimated number of IDU and sharing events

During the risk behaviour interviews, subjects were asked to estimate the frequencies of IDU and of sharing during the designated interval being queried to allow coding of frequency intervals. For the prospective cohort, sharing was designated by a positive response to the query, ‘Since your last interview, did you use injecting equipment after someone else had used it?’ and the frequency by, ‘Since your last interview, how often did you use equipment after someone else had used it?’. Both queries were coded as one of the following: ‘never’, ‘less than monthly’, ‘monthly or more’, ‘weekly or more’, ‘daily’, ‘more than daily’. For the current analysis, these estimates were converted into numbers of events per week using three different assumptions, labelled ‘conservative’, ‘intermediate’ and ‘progressive’, where the assumed number of events per category was increased from conservative to progressive, such as 1 per week up to 6 per week for ‘weekly or more’ (see Table 2).

**Table 2:** Numerical quantification (in times per week) of the injecting and sharing events.

A					
Prospective cohort					
	Less than monthly	Monthly or more	Weekly or more	Daily	Daily or more
**Conservative**	0.1/week	0.29/week	1/week	7/week	8/week
**Intermediate**	0.15/week	0.58/week	3.5/week	7/week	11/week
**Progressive**	0.21/week	0.87/week	6/week	7/week	14/week

B					
Retrospective cohort					
	Never	Sometimes	Most times	Always	
**Conservative**	0%	10%	60%	100%	
**Intermediate**	0%	25%	75%	100%	
**Progressive**	0%	40%	90%	100%	

Panel A. Numerical quantification (in times per week) of the injecting (both cohorts), and sharing events from categorical answers of the prospective cohort, provided by subjects during the HITS-p interviews to the questions “Since the last interview, how often did you inject drugs?” and “Since the last interview, how often did you use injecting equipment after someone else had used it?”. Panel B. Numerical quantification (as a percentage of the number of IDU events) of the sharing events in the retrospective cohort, based on the question “Since last imprisonment, how often did you use equipment after someone else had used it?”.

For the retrospective cohort, the number of injecting events was calculated as for the prospective cohort, from identical coded responses to the same question. However, for the number of sharing events a different set of coding categories was available, namely ‘never’, ‘sometimes’, ‘most times’, ‘always’. This was interpreted as 0%, 25%, 75% and 100% of the estimated injecting episodes respectively (two types of sensitivity analysis were performed; see below). The estimates of the absolute number of events in the period being queried were therefore obtained by multiplying the number of events by the length of the time-period (i.e., the time between the last interview and either the estimated date of infection or the date of the interview, for the prospective cohort; for the retrospective cohort: from entry into prison to either estimated date of infection, or to baseline interview date) in which these events had occurred.

### Maximum likelihood estimate (MLE) for per-event probability of infection

Data manipulation and statistical analyses were performed in the statistical package R v2.15.00 [13]. A maximum likelihood approach was used to estimate: i) the average per-event probability of infection during an episode of IDU with sharing of injecting equipment (with unknown infectious status of the source); and ii) the average transmission probability in the population with varied assumptions of the rate of actual contamination of the injecting apparatus with HCV. The estimate of the per-event infection probability (denoted *β* in equation 1) were obtained by maximising the likelihood function:
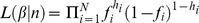
where *h_i_* = 1 if subject *i* became infected and 0 otherwise, and
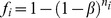
the probability that subject *i* became infected during *n_i_* episodes of IDU or sharing. A simulation-based analysis was performed to obtain a probability distribution for estimates of the per-event probability. Briefly, we simulated 10,000 random sub-samples (see [14] for a similar approach), and in each of these, ‘*n*’ episodes were randomly sampled (with replacement) from the pool of the total episodes of sharing. Similarly, the transmission probability was inferred by multiplying the number of sharing events *n_i_* by the assumed prevalence of HCV-contaminated injecting equipment. Simulations were performed in Matlab (R2011b, MathWorks, Natick, MA).

### Sensitivity analysis on sharing frequency

To assess the impact of the discrepancy between the high number of reported IDU events and the low number of reported sharing events (i.e., assuming under-reporting of sharing), the number of reported IDU events assumed to be associated with sharing was increased progressively from 15% to 100%. On each of these datasets, the MLE analysis was performed as described above. For this analysis, only the ‘intermediate’ quantification of the risk events (see above) was used to estimate the number of IDU events.

### Sensitivity analysis on the sharing estimate in the retrospective cohort

We estimated the number of sharing events by interpreting ‘always’, ‘most times’, ‘some times’ and ‘never’ as 100%, 75%, 25% and 0% of the estimated number of IDU events. This is the ‘intermediate’ scenario listed in Table 2b. Other scenarios were also tested and the results are in Figure S1. A second sensitivity analysis was performed; see Text S1 and Figure S2.

Groupwise comparisons of the risk behaviour of incident cases and uninfected subjects were performed with a Mann-Whitney test with continuity correction.

## Results

**Figure 1:**
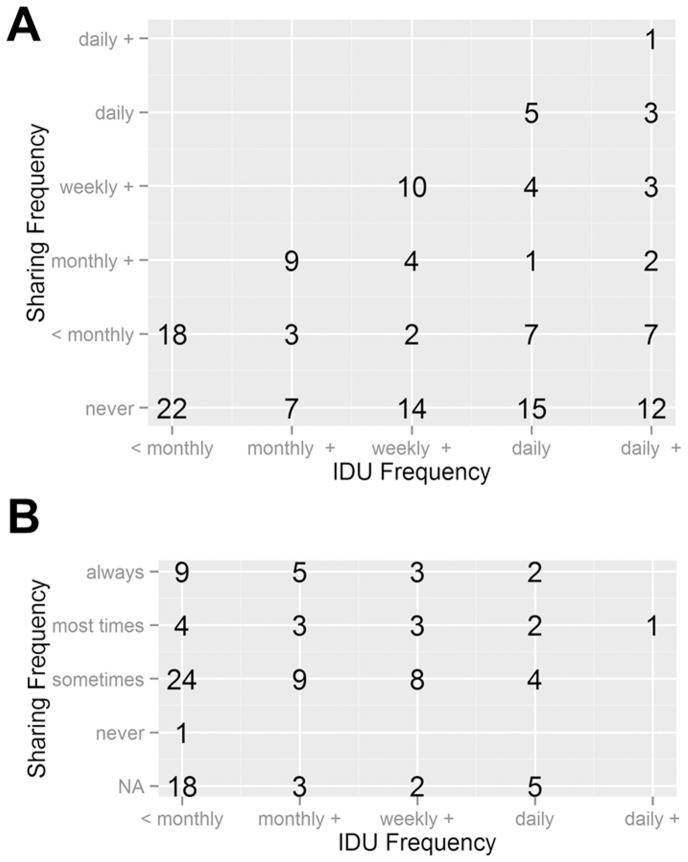
Number of interviews with self-reported injecting and sharing behaviours in the prospective (A) and retrospective (B) cohorts.

The prospective dataset included 92 subjects, who were predominantly male (N = 64; 70%), had a median age of 25 years (range 18 to 39 years), and had a median duration of IDU of 6 years (range 1 to 22 years). Table 1 summarises the demographic and risk behaviour characteristics of these subjects. There were 193 interviews available, corresponding to a total of 10,197 weeks of reported IDU and sharing events (Figure 1A). Sixty interviews (31%) recorded subjects reporting IDU daily or more, while in 79 (53%) interviews, there was at least one sharing event reported (Figure 1A). Almost all the IDU events involving sharing, include needle/syringe sharing (95%), while 45 (78%) of these also involved sharing of any element of the injecting paraphernalia. The median number of injecting episodes reported annually was 148 (IQR: 62–413) whereas the number of reported episodes of sharing of the injecting apparatus was 27 (IQR: 5–83) per year (excluding subjects who did not report sharing, both based on the intermediate estimates). During the two years of follow-up, 37 subjects became infected with HCV (40%), and all infected subjects reported IDU prior to the incidence time point; 22 of them also reported sharing during this period. A comparison of the risk behaviour of incident cases and uninfected subjects revealed a significantly higher number of IDU and sharing events among those that became infected (p<0.01 for IDU; p<0.05 for sharing).

**Figure 2:**
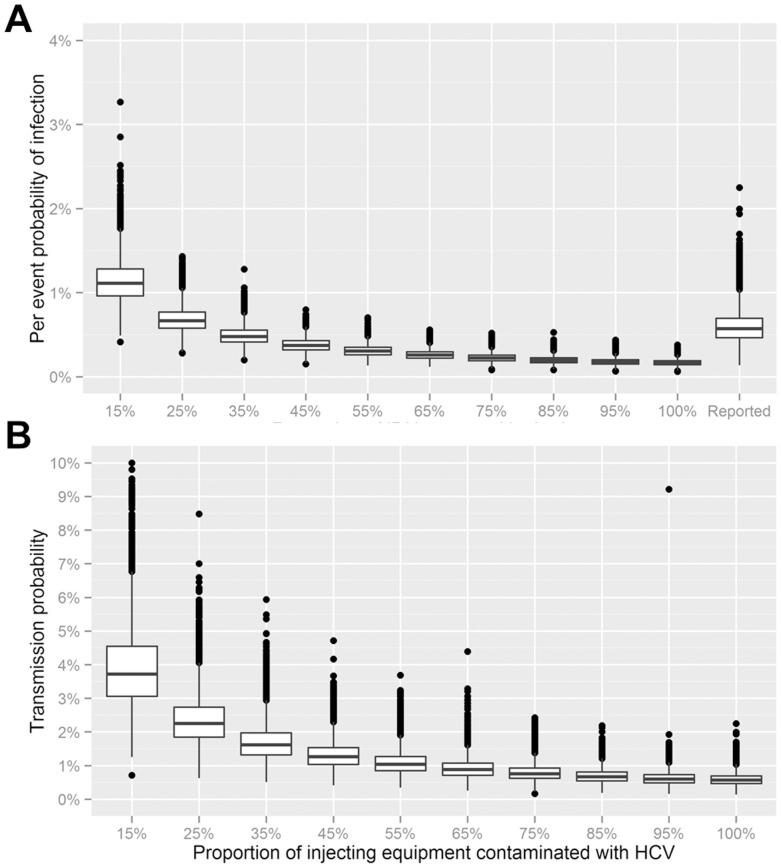
A. Sensitivity analysis to assess the effect of under-reporting of sharing during IDU. Modelling of the effect of under-reporting of sharing during IDU on the estimate of the per-event probability of infection during IDU with shared equipment. On the x-axis, the proportion *s* of IDU events that are also sharing events is represented. Boxplots represent the simulation-based distribution of the MLE for the estimate (for comparison, the actual result from the reported sharing distribution in the cohort is also reported). B. Boxplots representing the distribution of the MLE for the transmission probability in the population, as a function of the assumed proportion injecting equipment actually contaminated with HCV.

The estimate of the per-event probability of HCV infection after IDU following a sharing event (*β*, see equation 1 in Materials and Methods) is 0.57% (CI: 0.32%–1.05%, see Table 3). Notably, higher values were obtained when a conservative estimate of the number of risk behaviour events was assumed from the categorised period prevalence data from the interview. As a sensitivity analysis, the MLE estimates were repeated on simulated data where the number of IDU events that involved sharing, *s*, was assumed to vary between 15–100%. This revealed a substantial variability in the estimates for low values of *s* (<20%), whereas higher *s* values led to tighter estimates. The actual data for reported sharing corresponded to a scenario of around *s* = 30% (labelled “Reported” in Figure 2A). The estimate associated with the assumption that all IDU events were actually associated with sharing was 0.17% (95% CI: 0.11%–0.25%).

**Table 3:** Maximum likelihood estimate of the per-event probability of HCV infection during IDU events with sharing of injecting apparatus.

	Conservative	Middle	Progressive
**Prospective cohort**			
MLE estimate	1.00%	0.57%	0.40%
Median*	1.02%	0.57%	0.40%
95% CI*	0.53%–2.20%	0.32%–1.05%	0.23%–0.72%
**Retrospective cohort**			
MLE estimate	0.56%	0.42%	0.32%
Median*	0.56%	0.42%	0.32%
95% CI*	0.21%–2.5%	0.17%–1.48%	0.13%–0.99%

* Estimated from bootstrap analysis using 10,000 simulations (see Materials & Methods).

An estimate of the transmission probability (i.e., the probability of infection during a sharing event with contaminated injected apparatus) can be derived from the per-event probability of HCV infection and the proportion of shared equipment contaminated with HCV. The transmission probability is highly dependent on the prevalence of viraemia in the source(s) with whom sharing occurs, and blood contamination of the needle and syringe, as well the use and effectiveness of cleaning strategies such as bleaching. A sensitivity analysis was performed to assess the effect of variations in the proportion of shared equipment contaminated with HCV (ranging from 10% to 90%) on transmission. The increase in the prevalence of viral contamination of the shared injecting apparatus corresponded to a gradually decrease in the estimates of the transmission probability in the range of 0.5–6% (Figure 2B).

The per-event probability of HCV infection following a sharing event was estimated using the same approach in the retrospective dataset (N = 106). This analysis included data from 75 males (71%), with a median age of 24 years (range 18 to 48 years), and a median duration of IDU of 6 years (range 0 to 34 years). Table 1 summarises the subject characteristics. In this group, 77 (73%) subjects reported sharing events (Figure 1B), and 63 (82%) reported doing so only after bleaching the shared equipment. Of these, 99% reported sharing needle/syringe, and 75% reported sharing other components of the paraphernalia. The mean number of injecting episodes reported annually was 83 (median 6, range 6–572), coinciding with more than half of the subjects reporting IDU less than monthly, whereas the mean number of reported episodes of sharing of the injecting apparatus was 47 (median 8, range 2–429) per year.

There were 15 subjects who became infected with HCV between imprisonment and enrolment into HITS-p (14%), of whom 10 reported difference in the risk behaviour of incident cases and uninfected subjects (p>0.5 for IDU; p>0.5 for sharing). The MLE estimate for this group was 0.42% (0.17%–1.48%), comparable to the prospective dataset (Table 3). The MLE analysis was repeated on simulated data where the number of IDU events that involved sharing, *s*, was assumed to vary between 15–100% (Figure S1 B). The actual data for reported sharing corresponded to a scenario of *s* = 55%, whereas the MLE estimate for the per-event probability of HCV infection was 0.26% (0.13%–0.50%) if all IDU events were assumed to be associated with sharing.

Similarly, a second sensitivity analysis was performed to assess the effect of re-coding the quantitative categorical data regarding the number of IDU and sharing events on the estimated per-event probability of HCV infection. Nine scenarios were considered, corresponding to all possible combinations of conservative, intermediate and progressive values for both IDU and sharing events (Figure S1A, Table 2). Overall, the MLE estimates remained within a range of 0.26%–0.72%, with 95% CIs in the range 0.11%–3.8%. A higher estimate of the number of IDU events (progressive) corresponded to a gradual decrease in the MLE estimates. On the other hand, MLS estimates were higher when the number of IDU events involving sharing was assumed within the conservative range.

A third sensitivity analysis was also performed to assess the effect of the estimates for the proportion of IDU events involving sharing of injecting apparatus from the four categories ‘never’, ‘sometimes’, ‘most times’, ‘always’ (initially interpreted as 0%, 25%, 75% and 100% of the IDU event count, see Methods). In this analysis (Text S1), we have considered a range rather than a fixed proportion for each of these categories (excluding ‘never’ which was kept to 0%). This analysis revealed there was only a minimal effect on the estimate of the per-event probability, and as expected the confidence intervals increased with the broader range of values assumed for the number of sharing events (Figure S2).

## Discussion

This analysis provides, to our best knowledge, the first quantitative estimates of the per-event probability of HCV infection following an IDU event with sharing of injecting equipment (0.57%, 95% CI: 0.32%–1.05%), and of the transmission probability (0.5–6.0%) dependent on the assumed prevalence of HCV-contaminated injecting equipment.

The majority of existing estimates (Table S1) of the per-event probability of HCV infection relate to healthcare settings with needle stick injury (NSI). Within these reports, the number of NSI events within the cohorts is often incompletely recorded, and the associated source(s) generally only include those with confirmed HCV antibodies (i.e. sources with unknown status are not included). Furthermore, the viraemic status of the source(s) was recorded in only very few studies. Thus, these estimates are most comparable to the estimate of the transmission probability in the population reported here, as it is reasonable to assume that the majority of antibody positive NSI source subjects are indeed viraemic. Nevertheless, the pooled transmission probability estimate of HCV infection associated with NSI calculated from the published reports was 0.63% (95% CI: 0.5%–0.8%; reported range 0%–10%, Table S1), which is in the lower ranges of the transmission probability associated with IDU and sharing that is reported here. Of note, in the NSI setting, the depth of injury is an independent predictor of HCV transmission [15], [16]. A possible explanation for this difference in transmission probability could be related to significant injury caused by re-used and dull needles during an IDU act; especially in prison, where injecting drugs remains illegal. However, our estimates – as well as those based on NSI - are characterised by large confidence intervals, likely driven by a number of unresolved factors that could influence the estimates. For instance, the common lack of information regarding the viraemic status of the source, or the time-interval between the transmission event and the follow-up interview timepoint where infection was detected (up to 6 months, and up to 12 months in the two datasets reported here). Risk behaviours may also vary over these time periods, therefore our estimates should be regarded as average values over this period of time. These average estimates also do not account for heterogeneity in the type of sharing contacts, and the contribution of specific injecting equipment (needle/syringe, other injecting paraphernalia).

The estimates of the per-event probability of infection presented here are generally consistent with those obtained via mathematical modelling analyses that are in the range 1–3% [17], [18]. In particular, despite different approaches, the lower range estimate reported here of approximately 1%, is consistent with previous modelling-based estimates that accounted for an empirically defined social networks among PWID [18]. In this previous analysis there was a similar estimate for the distribution of weekly sharing events, but the model allowed testing of the effect of a well-defined distribution of per-contact events among PWID in Melbourne, Australia. Furthermore, a simulation-based analysis of HCV transmission among PWID in Glasgow, Scotland, revealed a lower, but significant, contribution to HCV transmission derived from injecting paraphernalia as opposed to the needle/syringe [17]. In the current analysis, the vast majority of sharing events involved the needle/syringe (Table 1), as expected in a prison setting where injecting equipment is scarce - therefore it was not possible to dissect the contributions of specific elements of the injecting apparatus. A meta-analysis of the literature on HIV-1 infectivity revealed a range of transmission probabilities for transmission during parenteral exposure falling between 0.63% and 2.4%, with a median of 0.8% [19]. The estimates of the transmission probability of HCV reported here range from 0.5% to 6%, depending heavily on the percentage of injecting equipment that is contaminated during shared use. Accurate information regarding the contamination of the injecting equipment is needed to make conclusive comparisons between HIV and HCV infection via this route.

In the Australian custodial population where this study was based, among prisoners with a history of IDU, the seroprevalence of chronic HCV infection is approximately 40% amongst males, and 80% amongst females – of whom the majority are viraemic [4], [20] Hence, the prevalence of viraemic individuals from whom transmission may occur is large. Currently, the only available prevention strategies in this setting are MMT (or buprenorphine) to reduce the frequency of injecting events amongst opioid-dependent PWID, and bleach cleansing of the injecting apparatus to reduce the likelihood of transmission. In the data reported here, the majority of subjects reported recent heroin use (∼70%), whereas only a minority (∼20%) were receiving MMT. A previous report from this setting has demonstrated that adequate MMT (>60 mg) is associated with a reduction in IDU [21].

A previous survey of 161 PWID conducted in the custodial setting revealed that only 3% of subjects reported that no other person had used the injecting equipment before them in their most recent injecting event in prison, and only 35% reported having never used injecting equipment after another person [20]. Although it is possible that the number of sharing events reported here are genuine as the availability of clean injecting equipment may vary between different custodial centres and in different periods of time, these data suggest that in the current study, the rate of sharing during IDU recorded is likely to be an underestimate of the actual rate, and hence the estimated transmission rates are likely to be relative over-estimates. In fact, the per event probability estimates, based on the assumption that all IDU events involve sharing, are respectively three (prospective cohort) and two (retrospective cohort) times smaller than the per event probability estimates based on the self-reported number of shared events.

Laboratory studies have suggested that the per-event probability of infection is also likely to be affected by the half-life of HCV in the injecting apparatus. These experiments showed that the virus can survive beyond one day, but the viral titre decays over hours [10]. However, there is no real-world data supporting these laboratory simulations. In addition, the efficiency of bleach cleansing is likely to be relevant, as it has been shown that commercially available disinfectants can rapidly eliminate viable virus to undetectable levels on inanimate surfaces [9]. Although bleach is readily available in the custodial setting reported here, IDU is punishable. Hence, there is a high probability that the cleansing process is rapid and furtive – and therefore potentially inadequate, as is reflected by the high incidence rate in the cohort.

Our study is limited by the categorical nature of the questionnaire responses. We have assessed this issue by interpreting these responses in three different manners, leading to a conservative, progressive and ‘intermediate’ analysis of the data (see Results). Accordingly, the estimated risk of HCV infection was also estimated with a reduction in the number of categories to two, namely “weekly or more” and “less than weekly”. This re-classification did not significantly affect the estimates of the per-event probability (data not shown), thus reinforcing the robustness of the approach. Another limitation is the absence of data regarding episodes of men having sex with men, as this was not included in the questionnaire. However, the risk of sexual transmission of HCV is considered low, at least in the absence of HIV, and probably negligible when compared to the direct blood-to-blood contact occurring during sharing of needles [22].

Given the low per-event risk of infection, this analysis suggests that an important strategy to reduce HCV transmission would be to reduce the number of sharing events, in addition to employing strategies aimed at reducing the per-event risk (e.g., by bleaching). This may be accomplished by implementing needle/syringe exchange programs, intensive rehabilitation, bleach and other decontamination strategies, or by increasing coverage of opioid substitution programs. Comparable analyses are warranted in community-based cohorts, notably in settings where there is ready access to needle and syringe exchange in order to estimate the protective effects. These transmission estimates will inform planning and evaluation of prevention strategies for the HCV epidemic in both community and custodial settings.

## Supporting Information

Figure S1 Sensitivity analyses to assess the effect on the per-event probability of infection of the re-coding and of under-reporting of IDU sharing events in the retrospective cohort.
(TIF)

Figure S2 A second sensitivity analysis to assess the effect on the per-event probability of infection of the quantitative estimates of the number of sharing events from the categorical data of the retrospective cohort. The x-axis shows the seven scenarios described in the Table in the Text S1, where we translate the qualitative assessment of frequency of sharing into a range of rather than a fix number representing the proportion of IDU events involving sharing. For each of this seven scenario - from the narrower to the broader range of estimates - we simulated 1000 datasets with estimates of the number of sharing events for each subject, assuming the intermediate IDU estimate as shown in Table 1B. The estimates on the per-event probability shown on the y-axis indicate a broader but comparable range of values consistent with the larger range of estimates assumed for the number of sharing events (see Text S1 for details).
(TIFF)

Table S1 Estimates of HCV transmission risk via needle stick injury in health care settings.
(XLSX)

Text S1 Sensitivity analysis to assess the effect on the estimates of the per-event probability of the quantitative estimates for the proportion of IDU events involving sharing injecting apparatus in the retrospective cohort.
(DOCX)

## References

[1] Global Burden Of Hepatitis C Working Group (2004) Global burden of disease (GBD) for hepatitis C. J Clin Pharmacol 44: 20–29. 14681338 10.1177/0091270003258669

[2] Pouget ER, Hagan H, Des Jarlais DC (2012) Meta-analysis of hepatitis C seroconversion in relation to shared syringes and drug preparation equipment. Addiction 107: 1057–1065. 22168373 10.1111/j.1360-0443.2011.03765.x PMC3348401

[3] Hagan H, Pouget ER, Des Jarlais DC, Lelutiu-Weinberger C (2008) Meta-regression of hepatitis C virus infection in relation to time since onset of illicit drug injection: the influence of time and place. Am J Epidemiol 168: 1099–1109. 18849303 10.1093/aje/kwn237 PMC2727245

[4] Butler T, Lim D, Callander D (2011) National Prison Entrants' Bloodborne Virus and Risk Behaviour Survey Report 2004, 2007, and 2010.: Kirby Institute (University of New South Wales) and National Drug Research Institute (Curtin University).

[5] Macalino GE, Vlahov D, Sanford-Colby S, Patel S, Sabin K, et al (2004) Prevalence and incidence of HIV, hepatitis B virus, and hepatitis C virus infections among males in Rhode Island prisons. Am J Public Health 94: 1218–1223. 15226146 10.2105/ajph.94.7.1218 PMC1448424

[6] Butler T, Boonwaat L, Hailstone S, Falconer T, Lems P, et al (2007) The 2004 Australian prison entrants' blood-borne virus and risk behaviour survey. Australian & New Zealand Journal of Public Health 31: 44–50. 17333608 10.1111/j.1753-6405.2007.00009.x

[7] Hunt DR, Saab S (2009) Viral hepatitis in incarcerated adults: a medical and public health concern. Am J Gastroenterol 104: 1024–1031. 19240708 10.1038/ajg.2008.143

[8] Skipper C, Guy JM, Parkes J, Roderick P, Rosenberg WM (2003) Evaluation of a prison outreach clinic for the diagnosis and prevention of hepatitis C: implications for the national strategy. Gut 52: 1500–1504. 12970145 10.1136/gut.52.10.1500 PMC1773842

[9] Doerrbecker J, Friesland M, Ciesek S, Erichsen TJ, Mateu-Gelabert P, et al (2011) Inactivation and Survival of Hepatitis C Virus on Inanimate Surfaces. 204: 1830–1838. 10.1093/infdis/jir535 PMC3247810 22013220

[10] Paintsil E, He H, Peters C, Lindenbach BD, Heimer R (2010) Survival of Hepatitis C Virus in Syringes: Implication for Transmission among Injection Drug Users. 202: 984–990. 10.1086/656212 PMC2932767 20726768

[11] Teutsch S, Luciani F, Scheuer N, McCredie L, Hosseiny P, et al (2010) Incidence of primary hepatitis C infection and risk factors for transmission in an Australian prisoner cohort. BMC Public Health 10: 633. 20964864 10.1186/1471-2458-10-633 PMC2975656

[12] Page-Shafer K, Pappalardo BL, Tobler LH, Phelps BH, Edlin BR, et al (2008) Testing strategy to identify cases of acute hepatitis C virus (HCV) infection and to project HCV incidence rates. J Clin Microbiol 46: 499–506. 18032621 10.1128/JCM.01229-07 PMC2238141

[13] Team RDC (2012) R: A language and environment for statistical computing. Vienna, Austria: R Foundation for Statistical Computing.

[14] Jin F, Jansson J, Law M, Prestage GP, Zablotska I, et al (2010) Per-contact probability of HIV transmission in homosexual men in Sydney in the era of HAART. AIDS 24: 907–913. 20139750 10.1097/QAD.0b013e3283372d90 PMC2852627

[15] Yazdanpanah Y, De Carli G, Migueres B, Lot F, Campins M, et al (2005) Risk factors for hepatitis C virus transmission to health care workers after occupational exposure: a European case-control study. 41: 1423–1430. 10.1086/497131 16231252

[16] Tomkins SE, Elford J, Nichols T, Aston J, Cliffe SJ, et al (2012) Occupational transmission of hepatitis C in healthcare workers and factors associated with seroconversion: UK surveillance data. J Viral Hepat 19: 199–204. 22329374 10.1111/j.1365-2893.2011.01543.x

[17] Corson S, Greenhalgh D, Taylor A, Palmateer N, Goldberg D, et al (2013) Modelling the prevalence of HCV amongst people who inject drugs: an investigation into the risks associated with injecting paraphernalia sharing. Drug Alcohol Depend 133: 172–179. 23791029 10.1016/j.drugalcdep.2013.05.014

[18] Rolls DA, Daraganova G, Sacks-Davis R, Hellard M, Jenkinson R, et al (2012) Modelling hepatitis C transmission over a social network of injecting drug users. J Theor Biol 297: 73–87. 22185979 10.1016/j.jtbi.2011.12.008

[19] Baggaley RF, Boily MC, White RG, Alary M (2006) Risk of HIV-1 transmission for parenteral exposure and blood transfusion: a systematic review and meta-analysis. AIDS 20: 805–812. 16549963 10.1097/01.aids.0000218543.46963.6d

[20] Indig D, Topp L, Ross B, Mamoon H, Border B, et al.. (2010) 2009 NSW Inmate Health Survey: Key Findings Report. Sydney.: Justice Health.

[21] Dolan KA, Wodak AD, Hall WD (1998) Methadone maintenance treatment reduces heroin injection in New South Wales prisons. Drug Alcohol Rev 17: 153–158. 16203480 10.1080/09595239800186951

[22] Tohme RA, Holmberg SD (2010) Is sexual contact a major mode of hepatitis C virus transmission? Hepatology 52: 1497–1505. 20635398 10.1002/hep.23808

