# Supplementary figures and images for: Per-Event Probability of Hepatitis C Infection during Sharing of Injecting Equipment
Source: PLoS One. 2014 Jul 7;9(7):e100749. doi: 10.1371/journal.pone.0100749 (PMC4085033; doi:10.1371/journal.pone.0100749)

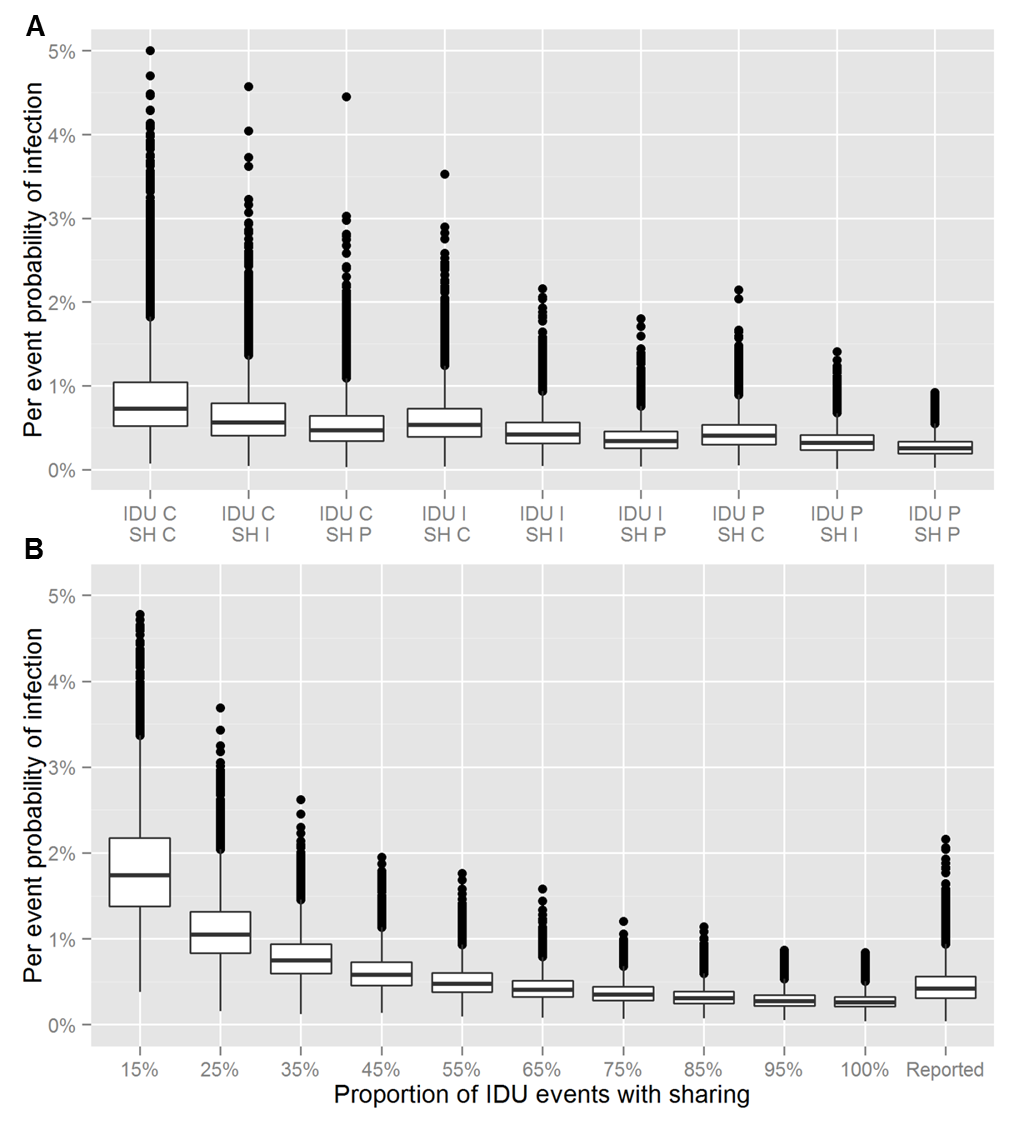

Supplement: Figure S1 — Sensitivity analyses to assess the effect on the per-event probability of infection of the re-coding and of under-reporting of IDU sharing events in the retrospective cohort. (TIF) [file pone.0100749.s001.tif]

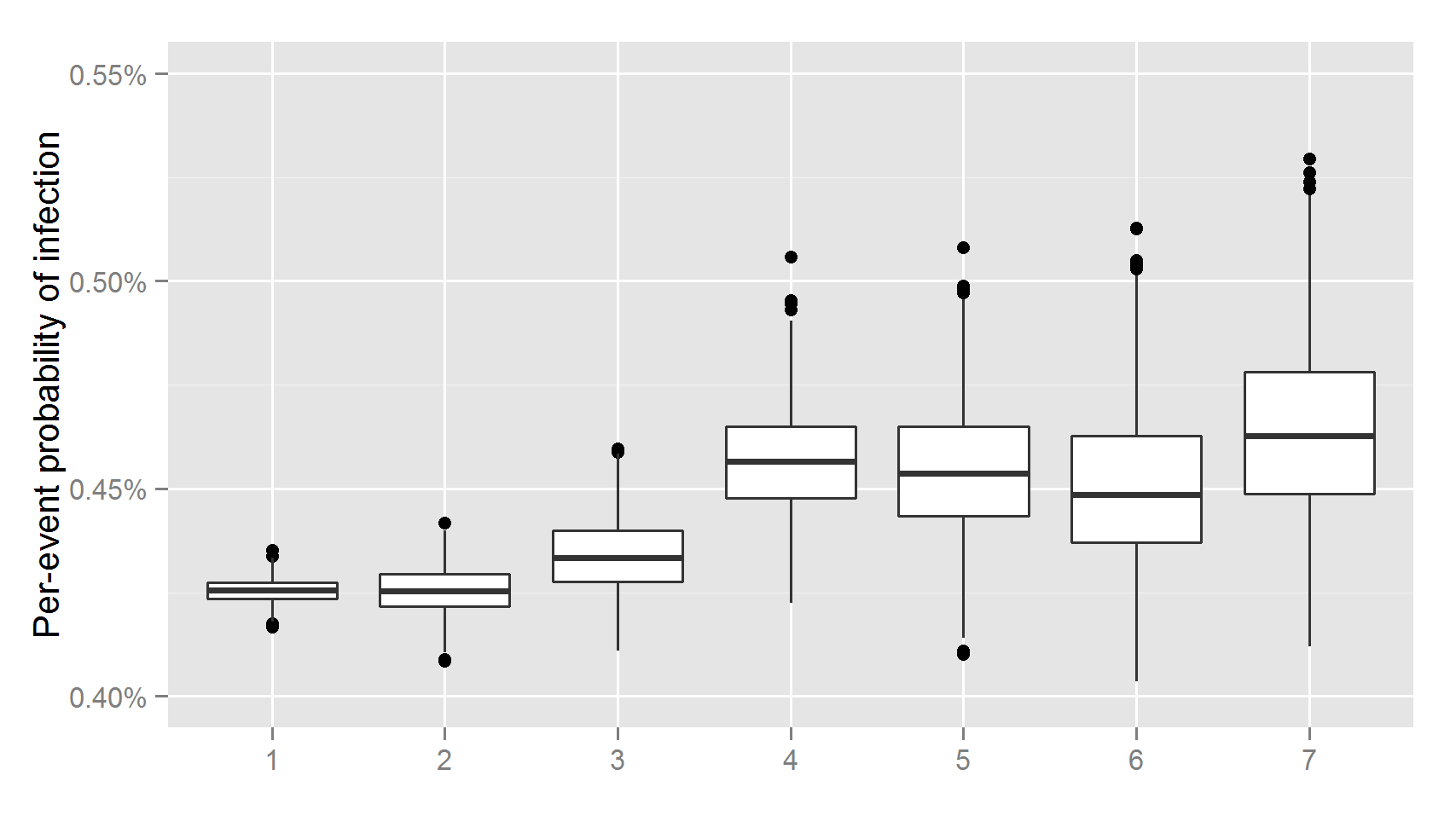

Supplement: Figure S2 — A second sensitivity analysis to assess the effect on the per-event probability of infection of the quantitative estimates of the number of sharing events from the categorical data of the retrospective cohort. The x-axis shows the seven scenarios described in the Table in the Text S1, where we translate the qualitative assessment of frequency of sharing into a range of rather than a fix number representing the proportion of IDU events involving sharing. For each of this seven scenario - from the narrower to the broader range of estimates - we simulated 1000 datasets with estimates of the number of sharing events for each subject, assuming the intermediate IDU estimate as shown in Table 1B. The estimates on the per-event probability shown on the y-axis indicate a broader but comparable range of values consistent with the larger range of estimates assumed for the number of sharing events (see Text S1 for details). (TIFF) [file pone.0100749.s002.tif]
