# Supplementary material for: Per-Event Probability of Hepatitis C Infection during Sharing of Injecting Equipment
Source: PLoS One. 2014 Jul 7;9(7):e100749. doi: 10.1371/journal.pone.0100749 (PMC4085033; doi:10.1371/journal.pone.0100749)
Supplement: Text S1 — Sensitivity analysis to assess the effect on the estimates of the per-event probability of the quantitative estimates for the proportion of IDU events involving sharing injecting apparatus in the retrospective cohort. (DOCX) [file pone.0100749.s004.docx]

**Text S1**

*Sensitivity analysis: interpreting the effect on the estimate of the per event probability of infection due to the estimate of sharing frequencies from categorical data in the retrospective cohort.*

In the retrospective questionnaire, subjects were asked to rate their IDU as either ‘never’, ‘less than monthly’, ‘monthly or more’, ‘weekly or more’, ‘daily’, ‘more than daily’. As a follow-up question, they are asked to express how often they would share injecting equipment during IDU. The answers were to be chosen from ‘never’, ‘sometimes’, ‘most times’, ‘always’. We interpreted these as 0%, 25%, 75% and 100% of the IDU event count.

To assess the validity of this strict definition, we carried out the following sensitivity analysis: instead of assigning a fixed percentage, we let the percentage vary within a certain interval. The intervals were chosen to be: broad, narrow or intermediate; each subject was assigned a percentage drawn randomly from these intervals, this independently from the other subjects who had given the same response to the sharing frequency question.

|  | Sometimes | Most times | Always |
| --- | --- | --- | --- |
| 1 | 20-25% | 75-80% | 95-100% |
| 2 | 20-30% | 70-80% | 90-100% |
| 3 | 15-30% | 70-85% | 85-100% |
| 4 | 10-30% | 65-85% | 80-100% |
| 5 | 10-35% | 60-85% | 75-100% |
| 6 | 10-40% | 55-85% | 75-100% |
| 7 | 5-40% | 50-85% | 75-100% |

For each of the seven scenarios in the table, we generated 1000 datasets with estimates of the number of sharing events for each subject, based on the intermediate IDU estimate. For these 7 x 1000 datasets, we computed the per-event probability of infection as described in the Method section (see Figure S2). We conclude there is little effect on the estimates.
